# Presence and characteristics of *senile pruritus* among Danish elderly living in nursing homes

**DOI:** 10.2144/fsoa-2019-0036

**Published:** 2019-06-24

**Authors:** Nanna Dyhre-Petersen, Parisa Gazerani

**Affiliations:** 1 Department of Health Science & Technology, Faculty of Medicine, Aalborg University, Aalborg, Denmark

**Keywords:** chronic itch, elderly, itch, nursing home, *senile pruritus*

## Abstract

**Aim:**

To explore the pattern of occurrence and characteristics of *senile pruritus* among elderly living in nursing homes in the Northern region of Denmark.

**Materials & methods:**

A Danish questionnaire was developed and distributed to six nursing homes from which 46 residents participated.

**Results:**

The prevalence of chronic itch was 28.9%. Evening–night and autumn–winter with an average daily itch of 30 min were reported. Itch interfered with night sleep and daily activities. Scratching was common with 61.5% accompanying scratch marks. Half of participants reported Xerosis. Cream, cold compress and cold shower were found to be the most effective remedies for itch relief.

**Conclusion:**

The present study revealed a high prevalence of chronic pruritus including cases of *senile pruritus* that needs further exploration for treatment or preventive strategies.

## Lay abstract

*Senile pruritus* is chronic itch of unknown origin among the elderly. The occurrence and characteristics among Danish nursing home residents was not known. We developed a Danish questionnaire and distributed it to 6 nursing homes, where 46 residents participated. We found a high prevalence of chronic itch (28.9%). Disturbing itch was reported in evening–night and autumn–winter. Itch interfered with night sleep and daily activities. Scratching was common with scratch marks. Showering precipitated itch, while cold shower was a reliever. Further studies are required to enhance the knowledge about senile pruritus and prevention or treatment strategies for elderly living in nursing homes.

*Senile pruritus* is the Latin term for itch that occurs among elderly. In order to clearly define *senile pruritus*, it is adroit to separate it in its two constituent parts, hence, ‘senile’ and ‘pruritus’. In this context, the word ‘senile’ refers to ‘one of old age’. No international agreement exists regarding an age limit for being categorized as one of old age, though commonly the limit is set to ≥60 years or ≥65 years [1]. Pruritus, which is frequently considered synonymously with the term itch [2,3], is defined as an unpleasant sensation that provokes the desire to scratch [4]. The International Forum for the Study of Itch (IFSI) recommends distinguishing itch as an acute or chronic itch in relation to its duration. Thus, an itch lasting less than 6 weeks is categorized as acute and lasting more than 6 weeks, as chronic [3,5]. Furthermore, an itch is classified according to etiology in six different categories: dermatologic diseases, systemic diseases, neurologic diseases, psychogenic/psychosomatic diseases, mixed and other (undetermined origin) [5]. The etiology of *senile pruritus* is unknown; however, current hypotheses are based on age-related changes in the skin and nervous and immune system [1]. Thus, *senile pruritus* is understood and defined as chronic itch of unknown origin in individuals of old age [1,5–7]. Compared with other medical conditions, only little attention has been given to *senile pruritus*, and generally to itches among elderly. A lack of attention has resulted in insufficient prevention and/or treatment as well as current treatment options being based on anecdotal approaches [6]. An itch is not a lethal condition, but it can be disabling and poses a high negative influence on the quality of life [8]. Concurrently, with the increasing life expectancy in many countries and increasing older population [9], it is relevant to explore and contribute to providing further knowledge about *senile pruritus*. During the past two decades several attempts in this field have resulted in a growing literature. Table 1 presents a summary of recent studies identified in the literature along with the reported prevalence of itch. Only two of the listed studies in Table 1 report a prevalence specific for *senile pruritus*. Both studies have been conducted in India and found a prevalence of *senile pruritus* of 10.6 and 9%, respectively [10,11]. However, these two studies did not elaborate on the distinction between pruritus and *senile pruritus*; hence, making the results less comparable with other studies. These studies clearly present a general problem within the research field of pruritus – namely, that pruritus is used as a broad term and not specified precisely [3]. This is further reflected in the studies in Table 1 that collectively have reported a prevalence of itch ranging from 2.5 to 79%. Additionally, some of the studies are based on a study population with the age of ≥0 years; hence, making it further difficult to assess how widespread a problem pruritus is among the elderly as well as revealing a pattern of occurrence and characteristics of *senile pruritus*.

**Table 1.** Prevalence of itch reported by different studies.

Study (year)	Year	Country	Sample size (age of study population)	Prevalence of itch (%)	Ref.
Kiellberg, Larsen and Sand (2005)	2003	Denmark	n = 428 (≥0 years)	2.5	[12]
Kiliç *et al.* (2008)	2006	Turkey	n = 300 (>75 years)	10.3	[13]
Yalçin *et al.* (2006)	1999–2003	Turkey	n = 4099 (≥65 years)	11.5	[14]
Bilgili *et al.* (2012)	2007–2010	Turkey	n = 5961 (≥65 years)	8.8	[15]
Thapa *et al.* (2012)	2010–2011	Nepal	n = 330 (≥60 years)	7.3	[16]
Reszke *et al.* (2015)	2012	Poland	n = 198 (≥65 years)	34.8	[17]
Darjani *et al.* (2013)		Iran	n= 440 (≥60 years)	22	[18]
Gunalan *et al.* (2017)	2014–2016	Puducherry, India	n = 300 (≥60 years)	44	[19]
Chowdhury, Das and Roy (2016)	2016	India	n = 200 (≥60 years)	79 (in which 10.6% was *senile pruritus*)	[10]
Jindal (2016)	2012–2014	India	n = 1380 (≥60 years)	9 (*senile pruritus*)	[11]
Polat *et al.* (2009)	2005–2006	Turkey	n = 209 (≥65 years)	18.6 (CP)	[20]
Valdes-Rodriguez *et al.* (2015)	2013	Mexico	n = 302 (≥60 years)	25 (CP)	[21]
Kopyciok *et al.* (2016)	2011	Germany	n = 334 (22–65 years)	36.2 (in which 87.6 was CP)	[22]
Zirwas and Seraly (2001)	Not reported	USA	n = 50 (age not reported)	100 (CP), 22 (due to a systemic disease)	[23]
Sommer *et al.* (2007)	2000–2002	Germany	n = 263 (8–95 years)	100 (CP), 41.8 (due to a dermatological disease), 13.3 (due to systemic disease), 0.4 (due to neurological condition), 44.5 (unknown origin)	[24]
Ständer *et al.* (2015)	Not reported	Germany	n = 3116 (1–99 years)	100 (CP), 6.3 (CP of unknown origin)	[25]
Weisshaar *et al.* (2006)	Not reported	Germany/Uganda	n = 132/n = 84 (14–84 years/1–75 years)	8 (CP of unknown origin)/3.6 (CP of unknown origin)	[26]
Dalgard *et al.* (2004)	2000–2001	Norway	n = 18,770 (30–76 years)	8.4	[27]
Matterne *et al.* (2011)	2008–2009	Germany	n = 2540 (>18 years)	13.5 (CP) (in which 50.5 was of unknown origin)	[28]

CP: Chronic pruritus.

## Aim

Given the lack of studies regarding *senile pruritus* in Denmark, the aim of the present study was to investigate the pattern of occurrence and characteristics of *senile pruritus* among elderly living in nursing homes in the northern region of Denmark.

## Methods

### Study population & recruitment

**Figure 1.**
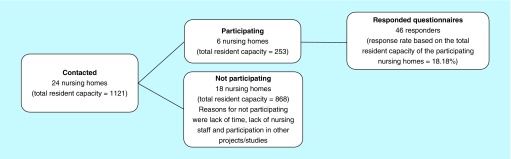
Flowchart of the recruitment of study participants.

A questionnaire-based study was conducted in the Aalborg commune in Northern Denmark during the period of September to December 2018. The study participants were recruited in October and November 2018 from public nursing homes located within the postal codes 9000 Aalborg, 9200 Aalborg SV, 9210 Aalborg SØ, 9220 Aalborg Øst, 9230 Svenstrup J, 9260 Gistrup and 9400 Nørresundby, all considered under the coverage of the Aalborg commune. The nursing homes were identified by consulting the publicly available list of nursing homes in Aalborg commune via the commune’s website [29]. In total, 24 nursing homes (total resident capacity = 1121) were identified and contacted by telephone in order to determine if the leader of the nursing home along with the nursing staff would cooperate in distributing the questionnaire to the nursing home residents. It was also considered that if necessary, help would be provided to the residents for filling out the questionnaire. The nursing staff was also asked to fill out a part of the questionnaire on behalf of the residents. Furthermore, the role of the nursing staff was to identify eligible residents who were mentally capable of understanding the questionnaire and who were not in such a severe physical state that they were bed-bound. Hence, the nursing home population was diverse in terms of functional and mental status, though all met the inclusion criteria. Six nursing homes (total resident capacity = 253) participated and 18 nursing homes (total resident capacity = 868) refused to join the study for a diverse range of reasons including lack of time, lack of nursing staff and participation in other projects/studies. Responders were in total 46 and the response rate based on the total resident capacity of the participating nursing homes was 18.18%. Figure 1 shows a flow chart of the study participants’ recruitment along with the total resident capacity and the total number of responded questionnaires, in other words, the sample size of the study. In addition, the response rate of the total resident capacity of the participating nursing homes is given.

It is important to mention that the resident capacity does not reflect the actual number of nursing residents. The capacity number has been chosen to be reported instead of the actual number because of the fluctuation in the number of actual nursing residents due to a continuously exchange of new and deceased residents. The nursing homes that expressed interest for participation in the study were given a period of 3 weeks to distribute the questionnaire among as many eligible residents as the nursing staffs had time to. The responded questionnaires were collected from the nursing homes after 3 weeks.

### Ethics

In accordance to the National Committee on Health Research Ethics guidelines, no ethical approval was required for conduct of this study questionnaire-based approach. All study participants were given information about the study. Informed consent was obtained from those participants who expressed interest freely in participation prior to answering the questionnaire. The information about the study was given by the nursing staff in accordance with the introduction stated at the beginning of the questionnaire. Eligible participants were clearly informed that the questionnaire was anonymous and that no sensitive personal data would be obtained about the participants. Furthermore, all nursing staff of the participating nursing homes were reminded by a written letter that no personal data were allowed to be passed on when the staff answered questions about medication and diagnoses on behalf of the participating residents.

### Questionnaire

A literature search was conducted in order to obtain an overview of existing literature about *senile pruritus*. The research was done in September 2018 by use of the database PubMed (US National Library of Medicine, NIH). The search was carried out using different search strategies. First, a so called ‘quick’ search was performed using the query phrase ‘senile pruritus’. This search led to few results and the acknowledgement of the need for additionally query words. Therefore, a second and more systematic search was carried out with the use of the following search words: ‘senile’, ‘elderly’, ‘aged’, ‘pruritus’ and ‘itch’. The words were combined in different ways to a single query by the Boolean Operators “AND” and “OR” and MeSH terms were used where possible. In order to narrow down the search in relation to the making of a questionnaire, the above query words were also searched for in combination with ‘questionnaire’, ‘diagnostics’ and ‘clinical practice’. Furthermore, the use of chain research was applied. English, Danish and German languages were included for the search. The results of the literature search were used as a basis for the development of a questionnaire, specifically for the detection and characteristics of *senile pruritus* among elderly at nursing homes in Denmark.

In making of the questionnaire for the present study, the following main criteria were used as a framework:

- The questionnaire should be designed for the elderly, in other words, a short questionnaire that is practicable for nursing home residents
- The questionnaire should be able to report basic demographic data
- The questionnaire should be able to detect *senile pruritus*
- The questionnaire should be able to capture relevant information about *senile pruritus*

To comply with these criteria, the results of the above-mentioned literature search were critically read and important findings concerning the main criteria were noted. Inspiration was also gathered from already existing itch severity scales and itch-related questionnaires and finally, a questionnaire consisting of 25 questions was designed. The first question was addressed to the nursing staff and concerned incidence of scabies among nursing staff and/or nursing home residents. Questions 2–6 asked the nursing staff to answer on behalf of the participating residents. These questions included age, gender, diagnoses, prescribed drugs and allergies. The reason for the nursing staff to answer these questions was that the staff has access to the residents’ medical journals, making it easy to print out a copy of this information as well as to ensure correct information about diagnoses and drugs. Question 7 asked residents whether they suffered from or had suffered from daily itch within the last year. Participants that answered ‘no’ to this question should not answer any more questions whereas participants that answered ‘yes’ should continue answering the remaining questions. The remaining questions (questions 8–25) concerned duration of itch, frequency of itch in 24 h, location, provoking and relieving factors, contact with animals, presence of skin rash, presence of xerosis (abnormally dry skin), awareness of scratching, scratch marks, specific situations that triggered the itch and own theory of the underlying cause of itch. Participants were also asked to evaluate the severity of itch, how much the itch affected daily activities, and night sleep. The evaluation was done according to a verbal rating scale (VRS) with 5 points. The temporal pattern of the itch was determined by asking of itch related to time of day and time of year. Additionally, the participants were asked if the itch was experienced as painful.

### Pilot study

A pilot study was conducted in October 2018 for identifying errors and omissions of the designed questionnaire. The questionnaire was given to six persons (three males and three females) at the age of ≥56 years who did not live in a nursing home. The test persons were instructed to complete the entire questionnaire even in the absence of suffering from itch. The persons were asked to comment on the questionnaire regarding errors and omissions. Based on the feedback from the test persons, the original questionnaire was revised for minor spelling errors and a small number of questions were added that were found useful for further clarification. Based on the pilot study, it was estimated that the questionnaire would not take more than 10 min to fill out.

## Statistical analysis

Data entry, storage and analysis were performed using the software IBM SPSS Statistics version 25 for Windows. A statistical significance level of 0.05 and 95% CI were used, in other words, stating statistical significance at the p < 0.05 level. The sample size of the study was 46, designated n = 46. All results with missing values were reported along with the number of valid and missing responders. Results generated from data with missing responders were reported based on the number of valid responders. Descriptive statistics for qualitative variables were given by number and percentages whereas quantitative variables were given as mean ± standard deviation (SD), or mode where appropriate. Association and differences between dichotomous variables were determined by Fisher’s Exact test due to the small sample size. Association between ordinal and dichotomous variables was done by use of Cochran–Armitage test of trend while differences were determined by Mann–Whitney U-test. Assumptions of the different tests were met, unless otherwise stated.

## Results

### Demographics & clinical characteristics

**Figure 2.**
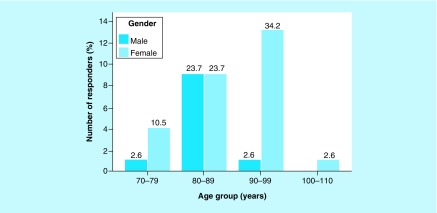
Bar chart of the numbers of males and females according to age groups. Percentage (%) within the total number of responders is shown on the top of each bar. n = 38, 8 missing.

The total number of responders was 46. All responders lived in 1 of the 6 nursing homes in Aalborg commune in Northern Denmark that had agreed to participate in the present study about *senile pruritus*. No cases of scabies within the recent 6 months were reported among any of the nursing home residents as well as the nursing staff (45 valid responders, 1 missing). Of the 46 responders, 14 were males (30.4%) and 32 were females (69.6%). The male-to-female ratio was 0.4. The mean ± SD age of the total number of responders was 87 ± 7.59 years. The minimum age was 71 years and the maximum age was 103 years (38 valid responders, 8 missing). The distribution of males and females according to age groups can be visualized in Figure 2.

In relation to a Mann–Whitney U*-*test, the distributions of scores of age groups between males and females were visually inspected by a population pyramid (not shown), which revealed that the distributions between males and females were not similar. The result of the Mann–Whitney U*-*test indicated no statistically significantly difference in the scores of age groups between males and females (U = 199; z = 1.771; p = 0.076).

Eight responders (17.8%; 45 valid responders, 1 missing) were reported to have one or more allergies (food, medicine, animals, pollen and/or perfume). Table 2 shows the distribution of males and females who had allergy. The difference between the proportion of males and females with existence of allergy was -14.2%. Due to small sample sizes, the Fischer’s Exact test was performed instead of the χ^2^ test for homogeneity. Fischer’s Exact test found no statistically significantly difference in the proportion of males and females with allergy (p = 0.405).

**Table 2.** Distribution of male and female responders according to existence of allergy and p-value for Fischer’s Exact test.

Allergy	Male (%)	Female (%)	Total (%)	p-value
No known	12 (92.3)	25 (78.1)	37 (82.2)	–
One or more allergies (food, medicine, animals, pollen, perfume)	1 (7.7)	7 (21.9)	8 (17.8)	0.405
Total	13 (100.0)	32 (100.0)	45 (100.0)	–

n = 45; 1 missing.

Among the 46 responders, a total of 165 diagnoses were identified within 27 different diagnostic groups (Table 3). This number is equivalent to an average of approximately four diagnoses per responder. Please note that the detected diagnoses are not equivalent to the number of diseases, in other words, a responder may have reported breast cancer and lung cancer, but the two diagnoses would only have been counted as one within the diagnostic group ‘cancer’. Likewise, 286 drugs were identified within 31 different groups of prescribed medicine, corresponding to an average of six drug groups per responder. Table 3 lists the frequency of diagnoses as well as prescribed drugs among the responders.

**Table 3.** Frequency of diagnoses and prescribed drugs reported by responders.

Diagnoses	Number of responses (% of total, n = 46)	Prescribed drugs	Number of responses (% of total, n = 46)
High blood pressure	18 (39.1%)	Vitamins and minerals	37 (80.4%)
Other diseases^†^	17 (37.0%)	Analgesics	31 (67.4%)
Vitamin, mineral and blood count deficiency (B12, D3, potassium, calcium, sodium, low hemoglobin)	15 (32.6%)	Antihypertensive drugs incl. nitrates and digoxin	28 (60.9%)
Diabetes I, diabetes II	12 (26.1%)	Anticoagulants	21 (45.7%)
Dementia	9 (19.6%)	Anticonstipation drugs	18 (39.1%)
Osteoporosis	9 (19.6%)	Proton pump inhibitors	16 (34.8%)
Rheumatic disease, incl. fractures	9 (19.6%)	Hypolipidemic drugs	13 (28.3%)
High cholesterol	9 (19.6%)	Diuretics	13 (28.3%)
Psychiatric disease	9 (19.6%)	Antidepressants	11 (23.9%)
Heart failure, arrhythmia	8 (17.4%)	Other drugs^‡^	9 (19.6%)
Stroke, heart attack	6 (13.0%)	Glucocorticoids	9 (19.6%)
Cancer	6 (13.0%)	Antidiabetic drugs	8 (17.4%)
Epilepsy	5 (10.9%)	COPD and asthma drugs	7 (15.2%)
Skin disease, psoriasis, xerosis, actinic keratosis, skin ulcer	5 (10.9%)	Anti-epileptics	6 (13.0%)
COPD	4 (8.7%)	Opioids	6 (13.0%)
Cataracts, glaucoma	4 (8.7%)	Alzheimer’s/dementia drugs	5 (10.9%)
Edema	4 (8.7%)	Antipsychotics	5 (10.9%)
Asthma	3 (6.5%)	Probiotics and extra nutrition	4 (8.7%)
Neuropathy	3 (6.5%)	Eye medication	4 (8.7%)
Ulcer	3 (6.5%)	Sex hormones	4 (8.7%)
Thyroid disease	3 (6.5%)	Antihistamines	4 (8.7%)
Hernia	2 (4.3%)	Uric acid – lowering drugs	3 (6.5%)
Constipation	2 (4.3%)	Urine bladder relaxants	3 (6.5%)
Ostomy	2 (4.3%)	Thyroid agents	3 (6.5%)
Ulcerative colitis	2 (4.3%)	Anti-osteoporotic drugs	3 (6.5%)
Urinary tract infection	2 (4.3%)	Antibiotic	3 (6.5%)
Kidney disease	2 (4.3%)	Antiparkinson drugs	3 (6.5%)
Total	173 (376.1%^§^)	Nicotine	2 (4.3%)
		Benign prostatic hyperplasia drugs	2 (4.3%)
		Cytostatics	2 (4.3%)
		Mucus relief	2 (4.3%)
		No drugs	1 (2.2%)
		Total	286 (621.7%^§^)

† The group ‘Other diseases’ is a compiled group of different diagnoses that only occurred a single time among the 46 responders. It consists of: headache, low bodyweight, pain in legs, leg cramps, leg restlessness, spinal compression fracture, enlarged prostate, gingivitis, dizziness, eye calcification, valve in the head, anemia, pleural exudate, bronchitis, incontinence, bladder prolapse, hemiparesis, Parkinson’s disease and arteriosclerosis.

‡ The group ‘other drugs’ is a compiled group of prescribed drugs that only occurred a single time among the 46 responders. It consists of: nonsteroidal anti-inflammatory drug, drug against diarrhea, hypnotic drug, insulin, caries prophylaxis, drug against leg cramps, unspecified liquid paraffin and psyllium husk.

§ Total number of reported items. The total number in percentage exceeds 100% because each responder was allowed to report more than one item. The total in % reflects the average of reported items per responder.

COPD: Chronic obstructive pulmonary disease.

19 responders (42.2%; 45 valid responders, 1 missing) stated to have experienced daily itch in the last few months or that they currently experienced daily itch. Of these, 5 responders (27.8%; 18 valid responders, 1 missing) reported itch lasting less than 6 weeks (i.e., acute itch) and 13 (72.2%) reported itch for more than 6 weeks (i.e., chronic itch). Out of the total number of responders, this corresponded to a prevalence of acute itch of 11.1% and to a prevalence of chronic itch of 28.9%, respectively. Table 4 shows the distribution of males and females with chronic itch together with the p-value of Fischer’s Exact test that shows that chronic itch is not dependent of gender (p = 1.00; 45 valid responders, 1 missing).

**Table 4.** Distribution of male and female responders defined as cases of chronic itch (itch lasting more than 6 weeks) and p-value for Fischer’s Exact test.

Cases defined as chronic itch	Males, n (%)	Females, n (%)	Total, n (%)	p-value
No	10 (71.4%)	22 (71.0%)	32 (71.1%)	–
Yes	4 (28.6%)	9 (29.0%)	13 (28.9%)	1.00
Total	14 (100.0%)	31 (100.0%)	45 (100.0%)	–

Characteristics of chronic itch. n = 45, 1 missing.

Two (15.4%) of the responders who had experienced chronic itch (hereafter designated as chronic itch [CI] responders), expressed it as a general itch affecting the whole body. The remaining 11 (84.6%) CI responders had in average 5.7 body parts where the itch was predominant. The body part that was most often affected by itch was reported as the upper anterior extremities and this was indicated by 7 (63.6%) CI responders. Shoulders, shoulder blades, chest and groin were the second most reported places, each reported by 6 (54.5%) CI responders. Stomach and lower posterior extremities were each stated by 5 (45.5%) CI responders and head, throat, back and lower anterior extremities were each affected in 4 (36.4%) of the cases. The least reported body parts were the back of the head, neck and buttocks as stated by 2 (18.2%) CI responders for each region. The upper posterior extremities, loin, anus and genitals were not reported by any of the 13 CI responders. Six (50%; 12 valid responders, 1 missing) stated that the average daily itch lasted longer than 30 min. For 3 (25%) CI responders, the itch was constant during the entire day. When asked what time of day (morning, noon, afternoon, evening, night) the itch was the most severe, evening and night were the most reported by 10 (76.9%) CI responders. The second most reported time of the day was afternoon by 7 (53.8%) CI responders. Morning and noon were reported by 3 (23.1%) and 2 (15.4%) CI responders, respectively. On average, the CI responders reported approximately two moments of the day where the itch was the most severe. Autumn was found to be the season where itch was reported as being the worst by 12 (92.3%) CI responders, closely followed by winter (11 [84.6%] CI responders). Spring and summer were reported by 2 (15.4%) of the CI responders, respectively. On average, two seasons were reported as being the time of year when the itch was most severe.

On a VRS from 1 (minimal itch) to 5 (maximal itch), the mode of severity of itch among the 13 CI responders was 2 on the scale, corresponding to the statement of the ‘weak itch’. Likewise, on two separate VRSs from 1 (no) to 5 (very strong), the interference of itch on night’s sleep and how disabling the itch was on daily activities, were reported by the CI responders. The mode of how much the itch interfered the CI responders’ night’s sleep was 2 and 3 on the scale corresponding to the statements of the ‘weak’ and ‘Moderate’ interfering of sleep. The mode of how disabling the itch was on daily activities was 2 and 3 corresponding to the ‘weak’ and ‘moderate’ disablement. Six (46.2%) of the CI responders perceived the itch as painful while the remaining 7 (53.8%) did not. According to a Cochran–Armitage test of trend that was run in order to find out if there was a linear trend between the reported severity of itch from 1 to 5 and the perceiving of itch as painful, no statistically significant linear trend (p = 0.330) was found. When asked about scratch marks, 8 (61.5%) of all CI responders reported to have marks.

**Figure 3.**
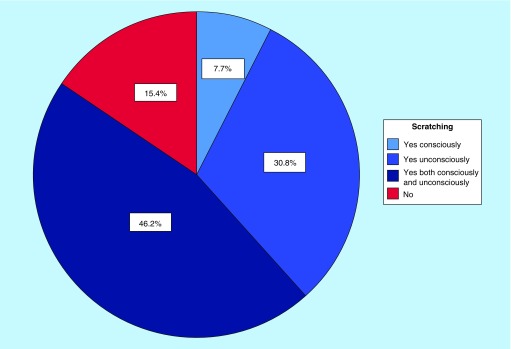
Pie chart of chronic itch responders’ scratching behavior. The chart shows the percentage of the total number of chronic itch responders, n = 13.

Figure 3 shows the CI responders scratching behavior. When asked about scratch marks, 8 (61.5%) of all CI responders reported to have marks.

### Possible underlying reasons for chronic itch

All CI responders were asked about animal contact since animals may be an originator of itch. None of the 13 (100%) CI responders had been in contact with animals.

Seven (53.8%) stated that the itch typically began in relation to showering or being in bed/resting. The remaining 6 (46.2%) did not experience itch in relation to a specific activity or situation. Also, 6 (46.2%) of the CI responders reported showering as an aggravating factor of itch while 1 (7.7%) stated heat (not further specified) as a factor. The remaining 6 (46.2%) did not report a factor that could worsen the itch.

Even though some of the CI responders could point out specific situations that initiated the itch as well as factors that exacerbated it, none of the 13 (100%) CI responders had an idea of the underlying cause of itch.

Five (50%) of the CI responders (10 valid responders, 3 missing) reported to have xerosis (abnormally dry skin) of the same body regions as where it was itchy. Two (15.4%) out of the 13 CI responders reported skin rashes at the same place where it was itchy, 1 (7.7) CI responder had skin rash in another place and the remaining 10 (76.9%) did not have any skin rashes. The p-values of Fischer’s Exact test were calculated for the cross tabulation of all the diagnoses, the prescribed drugs and the allergies reported by the 46 responders as compared with the CI responders. Table 5 presents the results with a statistically significant p-value.

**Table 5.** Distribution of cases defined as chronic itch according to diagnosis and prescribed drug together with p-value for Fischer’s Exact test.

Diagnosis or prescribed drug	Cases defined as chronic itch		Total (%)	p-value
	No (%)	Yes (%)		
Cancer, no	30 (93.8)	9 (69.2)	39 (86.7)	–
Cancer, yes	2 (6.3)	4 (30.8)	6 (13.3)	0.049
Total	32 (100)	13 (100)	45 (100)	–
Diuretics, no	26 (81.3)	6 (46.2)	32 (71.1)	–
Diuretics, yes	6 (18.8)	7 (53.8)	13 (28.9)	0.030
Total	32 (100.0)	13 (100.0)	45 (100.0)	–

Only results with p < 0.05 are listed in the table. n = 45, 1 missing.

### Relieving factors of chronic itch

All 13 CI responders were asked about relieving factors of their itch and 12 (92.3%) of them stated that cream, cold compress and/or a cold shower relieved the itch. Only 1 (7.7%) CI responder did not report any relieving factors.

## Discussion

In Denmark, the prevalence of pruritus has been found to be 2.5% in a study of Kiellberg Larsen and Sand [12]. This study was based on patients referred to an acute outpatient clinic in the age group of ≥0 years. We therefore attempted to identify presentation of *senile pruritus* in nursing home residents in the Aalborg commune by aid of a questionnaire that was constructed specifically to investigate the pattern of occurrence and characteristics of *senile pruritus*.

### Prevalence of itch

In the present study, a high prevalence of daily itch within the past year was found among 42.2% of the study population. This result is in sharp contrast to the findings by Kiellberg Larsen and Sand [12], which might be due to the higher age range of the present study population. Other studies with participants with the age ≥60 years or ≥65 years have in total reported a prevalence of itch ranging from 8.8 to 79% [10,13,15–19]. In relation to these findings, a prevalence of itch that increased with higher age has been reported among geriatric patients in a study by Yalçin *et al*. [14], as well as in a study among participants with the age ≥60 years conducted by Darjani *et al*. [30]. In Norway, a large survey study was performed by Dalgard *et al*. [27], which included participants in the age group of 30–76 years. This study also reported a higher prevalence of itch among older participants. In contrast to this, Matterne *et al*. [28], found a bimodal trend between age and lifetime prevalence of itch with peaks in prevalence for age groups 31–40 years and 51–60 years. Even though the existing literature about the prevalence of itch among the elderly varies a lot and that inconsistency rules regarding a linear connection between age and pruritus, the present result contributes to the acknowledgement of pruritus as a common issue among elderly.

### Prevalence of chronic itch

In the present study, 42.2% reported pruritus and among those, 72.2% categorized it as chronic itch, in other words, of the total study population, 28.9% had chronic itch. Furthermore, no statistically significant difference was found between males and females. These results are in line with those found by Kopyciok *et al.* [22], who reported that 87.6% of all pruritus cases could be categorized as chronic itch as well as no dependency of gender. Also, Polat *et al*. [20] and Valdes-Rodriguez *et al*. [21] reported a chronic itch prevalence of 18.6 and 25%, respectively. Despite no gender difference found in the present study, Bilgili *et al*. [15], found that itch was statistically more common among males than females. However, the study did not distinguish between acute and chronic itch, therefore leaving the possibility that the distinction has an influence on itch–gender interaction.

### Underlying causes of chronic itch

Neither scabies nor allergies were found to be likely causes of chronic itch in the present study, since no reports of scabies within the past 6 months were made by any of the responders’ nursing homes, and no statistically significant association was found between reported allergies and chronic itch. In contrast to this, having a diagnosis of cancer or being prescribed with diuretics were found to be statistically significantly associated with chronic itch (p = 0.049 and p = 0.030, respectively). Complaints of pruritus have frequently been reported among cancer patients and a twofold increased incidence of cancer among newly diagnosed pruritus patients have been found in a nationwide Danish cohort study [31]. In the present study, 30.8% of the responders with chronic itch were diagnosed with cancer. However, further studies must be carried out in order to reveal a causality. Diuretics have also been associated with itch either by directly causing itch or indirectly by inducing xerosis that can be pruritic [6,32,33]. Interestingly, Hayani *et al*. [34] found a lower prevalence of chronic itch in hemodialysis patients on loop diuretics; therefore, proposing a direct anti-inflammatory effect of loop diuretics that can attenuate itch. In the present study, 53.8% of the responders with chronic itch had been prescribed with diuretics. Furthermore, 50% of the responders with chronic itch reported to have xerosis. Since the present study did not investigate whether diuretics were the cause of xerosis, other explanations such as age-related changes in the skin’s barrier function must be kept in mind. Other potential underlying causes of chronic itch were indicated as showering or being in bed/resting, skin rash located at the same body regions as the itch, and heat as reported by 53.8, 15.4 and 7.7%, respectively. The reporting of showering as an activity that triggers itch may indicate aquagenic pruritus [35]. The presence of skin rash may also point to an underlying dermatologic disease causing chronic itch. Being in bed/resting under a duvet/blanket may increase the skin temperature. As reported in the present study, ambient heat has been found to aggravate itch and it has been proposed that heat increases itch sensation due to its effect on nerve endings [36]. It could be hypothesized that some individuals suffer from hypersensitive nerve endings, thus activities such as being in bed/resting results in itch, in other words, the itch may be of neurological origin. Collectively, the findings of cancer, diuretics, xerosis, aquagenic pruritus and skin rash as well as hypothesized neurological dysfunction may all serve as possible underlying causes of chronic itch in the present study. Therefore, the prevalence of chronic itch of 28.9% in the present study might only show that the exact prevalence of *senile pruritus* remains unknown, though most likely within this estimation.

### Characteristics of chronic itch

#### Location

In some cases, the itch location may indicate the underlying cause or distinguish it from other itch-related diseases. It may therefore be of great clinical value to reveal patterns of itch, although few studies have focused on this aspect [1]. Most of the responders with chronic itch in the present study reported a localized itch (84.6%). On average, approximately six body parts were affected by itch, with the upper anterior extremities (63.6%) as the most reported site. In contrast to this, two other studies by Valdes-Rodriguez *et al*. [21] and Kopyciok *et al*. [22] reported arm affected in 27 and 42.5% of the cases, respectively. Both studies found the lower legs were the most affected, by 56.6 and 54%, respectively. In addition to this, Valdes-Rodriguez *et al*. [21] also found that patients with chronic venous insufficiency experienced pruritus in the legs significantly more than patients without this condition. In the present study, the lower posterior extremities and lower anterior extremities were also reported, respectively by 45.5 and 36.4% of the responders. The least affected area reported in the studies by Valdes-Rodriguez *et al*. [21] and Kopyciok *et al.* [22] was the anogenital and genital areas. In the present study, no report was received on anogenital area being affected by itch. Despite some inconsistency, a pattern of locations affected by pruritus seems to exist with the larger body parts being the most affected and the anogenital the least affected. Variability in the results might be due to differences in the study populations. The study by Kopyciok *et al*. [22] was conducted among a random cohort of patients attending a general dermatology practice in Germany. The study by Valdes-Rodriguez *et al*. [21] was based on Hispanic geriatric subjects in Mexico. The present study, however, had a small study population of only 13 Danish nursing home residents. Identification of pattern of localized itch in future studies is warranted to investigate the sites of pruritus in relation to dermatomes in order to reveal a possible connection between pruritus and specific spinal innervation.

#### Daily & seasonal changes

The present study demonstrated that autumn and winter were the two seasons for occurrence of the most severe itch (92.3 and 84.6%, respectively), thereby contributing to an increasing literature of seasonal changes in itch. Likewise, Valdes-Rodriguez *et al*. [21] found that chronic pruritus in elderly was the most intense during winter. Seasonal changes in itch have been attributed to changes in air humidity. Dry, cold weather during autumn and winter may affect the skin barrier and has therefore been related to the aggravation of xerosis that again may aggravate itch. Beside seasonal changes, a growing literature on daily changes in itch has revealed a more pronounced itch at night [22,33]. The present study supports the existing literature on severity of itch in the late hours of the day and during the night. Nocturnal pruritus (itch at night) has been proposed to be a result of pathophysiologic alterations of key functions of the skin during sleep such as thermoregulation, maintenance of fluid balance and barrier function [37]. The circadian rhythm has also been proposed to play a role in nocturnal pruritus since the level of corticosteroids is low in the evening and night. The lower level of corticosteroids leads to a decreased anti-inflammatory response thereby inducing a pro-itch environment [38,39]. Furthermore, it has been hypothesized that dysregulation of central mechanisms is involved in nocturnal pruritus. This is based on the inhibitory action of the executive frontal lobe that is high during the day, thus preventing scratching behavior and enhancement of pruritus [37]. Additionally, daily activities can play a distracting role, hence the absent of other stimuli during the night increases the perception of itch [36].

#### Scratching

It is well known that itch is accompanied by a strong desire to scratch. The present study demonstrated a high prevalence of scratching with over half of the responders having scratch marks. Recent studies have shown that scratching reduces the activity in the primary somatosensory cortex (S1), which is associated with sensory-discriminative aspects of itch such as intensity, location, quality and duration. Hence, by scratching, one is capable of modulating the itch sensation and evoking itch relief. Furthermore, research has shown that scratching affects the brain’s reward circuits (especially the ventral tegmental area, substantia nigra and the raphe nucleus) as well as the prefrontal cortex, which demonstrated to play an important role in controlling motivation-based actions [40]. Collectively, this may explain why some individuals keep on scratching themselves even when it results in scratch marks. Subjects with chronic itch are therefore at risk for developing a harmful itch-scratch cycle that ultimately leads to development of skin lesions, further increasing the risk of itch due to breakdown of the skin barrier. The findings in the present study strongly indicate the need for successful treatment in order to prevent potential development of harmful scratching behavior.

#### Quality of life

It has been shown that chronic itch negatively influences the affected person’s daily life. Kopyciok *et al*. [21] found that 77.7% reported the daily itch as frequent to permanent. Likewise, in the present study, 50% reported the average daily itch as lasting for longer than 30 min. and 25% reported the itch as constant. This clearly indicates the importance of pruritus and its impact on the overall wellbeing. The aspect of quality of life is further proven in the present study by the results of the verbal rating scale. The mode of severity of itch was rated as ‘weak itch’ and the mode of itch’s interference on night’s sleep as well as daily activities were both rated as ‘weak’ and ‘moderate’. Similar results for itch-related sleeplessness and restriction in daily life have been found by Kopyciok *et al*. [22]. The burden of itch was also reported by Weisshaar *et al*. [26], who clearly found a negative impact on quality of life among patients with pruritus. Matterne *et al*. [28] found a significant correlation between itch dermatosis severity and quality of life as well as emotional wellbeing. This may indicate that the measurement of severity of chronic itch can be used as a reliable tool in the clinic when evaluating the success of a treatment strategy.

Furthermore, 46.2% of the responders in the present study perceived the itch as painful. Although no linear trend was found between severity of itch and painful perception of itch, the result indicates a relationship between itch and pain. The pathophysiology of pain and itch has come a long way, though no precise explanation can be given for why some individuals perceive itch as painful while others do not. The relationship with pain is not only seen concerning physiology but also in quality of life. It has been found that chronic pruritus has a quality of life’s impact comparable to that of chronic pain. It was also found that the average patient was willing to forfeit 13% of life expectancy to live without pruritus [8]. Collectively, chronic pruritus may carry a considerable burden and it should be taken into account seriously.

#### Relieving factors of chronic itch

Cream, cold compress and/or a cold shower were stated by 92.3% in the present study as a relieving factor of itch. Since many nursing home residents have a reduced capability of self-caring, this result is of beneficial use for nursing staffs in order to meet the residents’ needs and ensure their wellbeing. The result is furthermore in line with suggested symptomatic treatment options for the relief of itch as stated by the European Dermatology Forum (EDF) [3].

#### Study limitations

This study is not exempt of limitations. First, the sample size of the present study was rather small, thus making general conclusions must be with caution. Second, the questionnaire used in the study was constructed specifically for the study purpose since no validated questionnaire in Danish was available. Therefore, a pilot study was conducted in order to enhance the validity at the first step. Third, since many nursing home residents may not have the abilities and/or devices required to answer an online questionnaire, the study questionnaire was prepared in paper format. In this format, missing data could not be controlled in the same manner as if the questionnaire was digital. Fourth, self-report is subjected to biases such as recall bias and social desirability. Nevertheless, this method is useful for obtaining subjective experiences and frequencies of symptoms [41], in particular in the case of this study that was the first attempt in the Danish resident houses. Future studies must focus on cases of *senile pruritus* among chronic itch in elderly to highlight the putative role of similar or different elements in genesis of itch. It is also important to identify if other etiologies can cause similar symptoms to avoid misdiagnosis or delay accurate diagnosis and treatments.

## Conclusion

The present study revealed a high prevalence of pruritus, with ‘chronic itch’ being the most pronounced, among Danish nursing home residents in Northern Denmark. Several possible causes of chronic itch were found including cancer, diuretics, skin rash, xerosis, aquagenic pruritus and possible neurologic dysfunction. Collectively, the itch was characterized as affecting multiple body parts, lasting for a long time each day, being more pronounced in the evening and night as well as in the autumn and winter, having a negative impact on quality of life and leading to the development of scratch marks. Few but important factors such as cream, cold compress and/or cold shower were found to relieve the itch. Further studies should be conducted in order to identify cases of *senile pruritus* among those with chronic pruritus to enhance the knowledge of prevention and treatment for those elderly living in nursing homes with long lasting itch.

## Future perspective

*Senile pruritus* in elderly is relatively neglected. There is a need for awareness and identification of cases to assist with chronic itch relief or prevention strategies. In future trials, a clinical diagnosis should be implemented and *senile pruritus* must be characterized as a separate entity and guidelines should be specifically created for this phenomenon. Identification of elderly residents in nursing homes with this condition can help determine better treatment strategies that can enhance the quality of life and functioning of these individuals.

**Summary points**

- *Senile pruritus* is understood and defined as chronic itch of unknown origin in individuals of old age. Compared with other medical conditions, only little attention has been given to *senile pruritus*, and generally to itch among the elderly.
- Lack of attention to prevalence of *senile pruritus* has resulted in insufficient prevention and treatment.
- Based on the current literature, it is difficult to assess how widespread a problem* senile pruritus* is.

**Aim**

- To investigate the pattern of occurrence and characteristics of *senile pruritus* among elderly living in nursing homes in the northern region of Denmark.

**Methods**

- A questionnaire-based study was conducted in the Aalborg commune in northern Denmark during the period of September to December 2018.
- The questionnaire was created specifically for the study and was based on a comprehensive literature search on the research topic.
- Descriptive statistics were applied and differences as well as associations between data were further evaluated by Fisher’s Exact test, Cochran–Armitage test of trend and Mann–Whitney U*-*test due to small sample size.

**Results**

- In total, 46 residents participated (30.4% males; 69.6% females; mean ± standard deviation age of 87 ± 7.59 years).
- The prevalence of chronic itch was 28.9%, independent of sex.
- Average daily itch of 30 min or longer was reported by 75%.
- The most severe itch was reported to occur in evening–night and autumn–winter.
- Interfering of itch with night sleep and daily activities was most often described as ‘weak’ or ‘moderate’.
- Scratching was common (84.7%) with 61.5% accompanying scratch marks.
- Xerosis was indicated by 50%.
- Showering was found an itch precipitating factor (53.8%).
- Cream, cold compress and cold shower were found the most effective remedies for itch-relief.
- Cancer diagnosis (p = 0.049) and treatment with diuretics (p = 0.030) were both associated with chronic itch.

**Discussion & conclusion**

- *Senile pruritus* can exert a high negative influence on the quality of life of elderly population, which is the growing population at present.
- Providing further knowledge about *senile pruritus* is crucial to enhance awareness and facilitate setting up preventive or treatment plans. There are no specific guidelines available.
- There is a need for trials to specifically evaluate *senile pruritus* as an entity, which can therefore be utilized to create those preventive or treatment guidelines.
